# Near infrared-light responsive WS_2_ microengines with high-performance electro- and photo-catalytic activities†
†Electronic supplementary information (ESI) available: Supporting tables and figures and Video S1. See DOI: 10.1039/c9sc03156a


**DOI:** 10.1039/c9sc03156a

**Published:** 2019-10-28

**Authors:** Víctor de la Asunción-Nadal, Beatriz Jurado-Sánchez, Luis Vázquez, Alberto Escarpa

**Affiliations:** a Department of Analytical Chemistry, Physical Chemistry and Chemical Engineering , University of Alcalá , Alcala de Henares , Madrid , E-28871 , Spain . Email: beatriz.jurado@uah.es ; Email: alberto.escarpa@uah.es ; Tel: +34 91 8854995; b Chemical Research Institute “Andrés M. del Río” , University of Alcala , Alcala de Henares , Madrid , E-28871 , Spain; c Materials Science Factory , Institute of Materials Science of Madrid (ICMM-CSIC) , Cantoblanco , E-28049 Madrid , Spain

## Abstract

Tungsten disulfide (WS_2_)-based micromotors with enhanced electrochemical and photo-catalytic activities are synthesized using a greatly simplified electrochemical deposition protocol at room temperature involving exclusively tungstic acid and sulfate as metal and sulfur sources without further building chemistry. The WS_2_-based micromotors exhibit dual electrochemical and photo-catalytic behavior in the inner and outer layers, respectively, due to the combination of the unique properties of the sp^2^ hybridized WS_2_ outer layer with highly reactive WS_2_-induced inner catalytic layers, accounting for this material's exclusive enhanced performances. A rough inner Pt–Ni layer allows tailoring the micromotor propulsion, with a speed increase of up to 1.6 times after external control of the micromotor with a magnetic field due to enhanced fuel accessibility. Such a coupling of the attractive capabilities of WS_2_ with enhanced micromotor movement holds considerable promise to address the growing energy crisis and environmental pollution concerns.

## Abstract 2

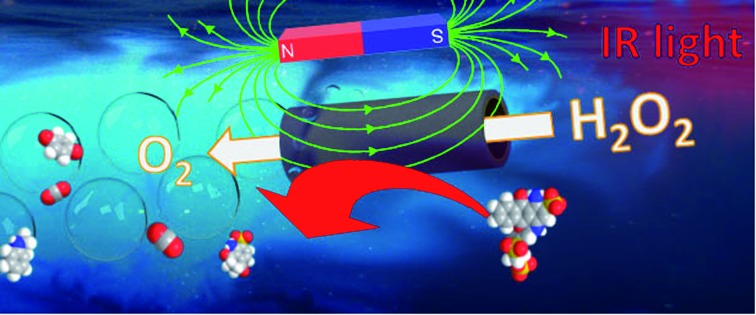
Tungsten disulfide based micromotors with enhanced electrochemical and photo-catalytic activities are synthesized using a simple electrochemical approach at room temperature without further building chemistry.

## Introduction

Transition metal dichalcogenides (TMDs)^1^,^2^ are an emerging type of two-dimensional (2D) materials consisting of a single layer of atoms. In particular, tungsten disulfide (WS_2_) displays a graphene-like structure in which each individual layer of W atoms is hexagonally packed between two trigonal atomic layers of S atoms. Such a structure offers 2D permeable channels for electron transport whereas a potential barrier along the vertical layers hinders electron jumping. This results in strong quantum confinement of electrons, which along the ultrahigh specific surface results in superior charge transfer.^3^,^4^ Such a unique combination of physical, optical, chemical and electronic properties has been exploited in a myriad of applications such as electronic devices,^5^ photocatalysis,^6^–^8^ energy storage,^3^,^9^–^11^ sensing^12^–^14^ and biomedicine.^15^–^17^ The fabrication of continuous WS_2_ films has been achieved by top–down exfoliation of the bulk material^18^ or bottom–up approaches such as chemical vapor deposition, hydrothermal synthesis or sulfurization.^18^–^20^ Electrochemical synthesis is a simple approach that can be performed under ambient conditions using aqueous precursors, yielding excellent nanostructures for (photocatalytic) processes.^21^–^23^

The field of micromotors has only recently started to benefit from the outstanding properties of TMDs. Micromotor design and the synthetic route adopted will exert a strong influence on the overall catalytic performance for each specific application.^24^–^31^ Thus, Pumera's group demonstrated the autonomous movement of naphthalene doped MoS_2_ nanoparticles that can be self-propelled *via* the combination of self-exfoliation and Marangoni effects.^32^ Later on, the same group incorporated WS_2_ nanoparticles onto the surface of polyaniline microrockets *via* template electrodeposition for *on-demand* delivery of the capacitive elements.^33^ TMDs can be used as a scaffold for building more complex structures exploiting their high surface area, as drug carriers or as sensing elements by modifying their surface with fluorescent molecules. Wang's group illustrated the use of template electrodeposited MoS_2_ micromotors for drug delivery and (bio)chemical sensing.^34^ In this latter application, their luminescence and photochemical properties make them promising candidates for further development of micromotors. However, despite the outstanding properties demonstrated by such early micromotor proof-of-concept applications, the design and applications of TMD micromotors still remain largely unexplored.

We report here, for the first time, the synthesis and application of micromotors with an *in situ* room-temperature electrodeposited WS_2_ external layer with dual “*inner and outer*” catalytic activities. To this end, we adopt a simplified electrochemical deposition technique at room temperature using tungstic acid and sulfate exclusively as sources for micromotor synthesis. Successful materials synthesis will be illustrated by scanning electron microscopy, atomic force microscopy and Raman characterization. We will also describe the dual “*inner and outer*” catalytic activities of the micromotors associated with the presence of a high density of edges and inherent photoinduced electron transfer (outer layer) and a rough catalytic patch (inner layer). The rough inner Pt–Ni layer allows tailoring of the micromotor propulsion, with a speed increase of up to 1.6 times after external control of the micromotor with a magnetic field due to enhanced fuel accessibility. The catalytic performance of the micromotors will be also illustrated for future “*on-the-move*” energy generation schemes. More interestingly, WS_2_ micromotors possess inherent NIR responsive properties, which will be exploited here for the enhanced removal of a model pollutant dye. Current trends in renewable energy utilization are aimed at the use of solar light for photocatalytic pollutant degradation and hydrogen generation *via* water splitting. Up to now, UV and visible light-triggered micromotors have been extensively studied for photodegradation processes,^35^–^37^ with NIR light only used for enhanced propulsion and triggered drug release schemes.^38^,^39^ Unlike such early efforts, the new micromotors described here hold considerable promise for the utilization of solar light for a myriad of renewable energy utilization schemes.

## Results and discussion

**Fig. 1:**
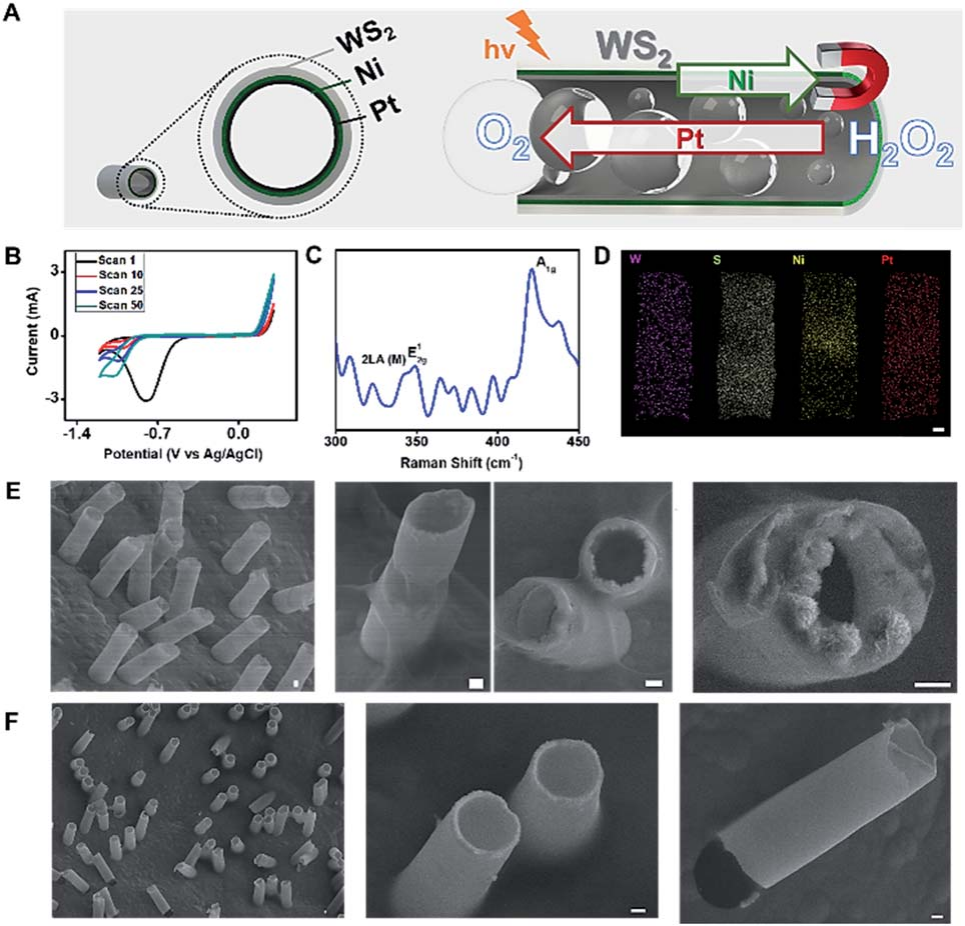
(A) Schematic of the NIR responsive WS_2_/Ni/Pt micromotors with electrochemical and photo “*inner and outer*” catalytic activities. Red arrow indicates the direction of oxygen bubble ejection by Pt decomposition whereas green arrow indicates micromotor direction after orientation with a magnet. (B) Cyclic voltammograms corresponding to the electrodeposition of WS_2_ micromotors. (C) Raman spectra; (D) EDX and (E) SEM characterization of the micromotors. (F) SEM images of control MoS_2_ micromotors. Scale bars, 1 μm.

Fig. 1 illustrates the preparation and characterization of the WS_2_ micromotors. For micromotor synthesis, we adopted a template electrodeposition protocol described by Wang's group.^34^ In this work, commercially available ammonium tetrathiomolybdate was used as the sole precursor for the facile synthesis of the MoS_2_ film by electropolymerization. However, for tungsten-based materials, the synthesis is more complex since thiol containing salts (such as S precursors) are required. In addition, the synthesis of ordered nanoporous metal sulfides is hindered by the uncontrollable fast precipitation between the metal and the S^2–^ ions.^21^ In order to overcome such problems, we designed a new strategy, based on a previous protocol, to control the reaction parameters and avoid precipitation and other unwanted chemical reactions, which is the main novelty here. Thus, deposition was carried out by cyclic voltammetry (CV) from a plating solution containing H_2_WO_4_ (0.135 M), Na_2_WO_4_ (0.135 M) and Na_2_SO_3_ (0.18 M) at pH 9.2 with a scan rate of 50 mV s^–1^ over the 0.3 to –1.2 V range (*vs.* a Ag/AgCl reference electrode for 10 cycles). As can be seen in the CV voltammogram in Fig. 1B, in the first scan (*n* = 1) a sharp increase in the cathodic current is observed at –0.78 V, which can be attributed to the fast generation of W^4+^ ions (due to the reduction of W^6+^ ions present in WO_4_^2–^) in the solution. As the potential cycling proceeds, a new reduction peak at –1.1 V can be observed, with an increase in the current along with the number of scans (from 5 to 50). This also indicates the reduction of W^6+^ ions present in WO_4_^2–^ to W^4+^, which subsequently reacts with the S^2–^ ions, generating a WS_2_ continuous thin film within the walls of the PC membrane. The electrodeposition mechanism can be described as follows:^21^W^6+^ + 2e^–^ → W^4+^W^4+^ + S^2–^ → WS_2_

Next, the effect of WS_2_ electrodeposition time was evaluated by varying the number of scans from 2 to 50 and using Pt as a second metallic layer. A low content of the material was electrodeposited after 2 scans, which resulted in ill-defined structures that collapse easily. Defined surface morphology and efficient propulsion in peroxide solutions were observed after 10 cycling scans. No noticeable differences were noted by increasing the number of scans; thus 10 scans were selected as optimal. The resulting electrically conductive WS_2_ continuous film provides an ideal template for the electrodeposition of magnetic (Ni) and catalytic (Pt) layers for further functionalization and autonomous movement in the intended applications, as will be described later.

To further support the proposed electrodeposition mechanism, Raman characterization was carefully performed. The WS_2_ crystalline structure is characterized by a trigonal prismatic arrangement of S–W–S atoms in which each plane contains a trilayer composed of an individual tungsten layer, sandwiched between two sulfur layers in trigonal prismatic coordination. Thus, such a structure displays characteristic optical phonon modes at the Brillouin zone center: E12g (in-plane optical mode), A1g (out-of-plane vibration of the sulfur atoms) and LA(M) (longitudinal acoustic mode at the M point or in-plane collective movements of the atoms in the lattice).^40^ The Raman spectra in Fig. 1C display the above-mentioned peaks: LA(M) at 330 cm^–1^, E12g at 350 cm^–1^ and A1g at 410 cm^–1^. According to the literature, this might be an indication that the material is present in the film as a mix of monolayer and bilayer structures.^41^ Overall, the Raman profile indicates the successful formation of the WS_2_ outer layer of the as-synthesized micromotors. This is fully supported by the energy-dispersive X-ray (EDX) spectroscopy mapping in Fig. 1D, which illustrates a uniform distribution of W and S along the resulting microtubes, with an inner Pt–Ni metallic layer. Top and cross-view scanning electron microscopy (SEM) images in Fig. 1E reveal the well-defined conical structure of the micromotors with a thin outer WS_2_ layer composed of granular particles with an average diameter of ∼190 nm. The geometry of the micromotors was characterized by observation of the SEM images of 50 micromotors. The average length was 11.4 ± 1.1 μm, with a thin outer Ni–Pt layer with an average thickness of 0.41 ± 0.24 μm and an average inner opening of 4.6 ± 0.3 μm. The micromotors are considered cylindrical, with two openings with radii *R*_j_. For comparison, Fig. 1F displays the SEM images of MoS_2_ micromotors prepared using a similar procedure to that described by Wang's group.^34^

**Fig. 2:**
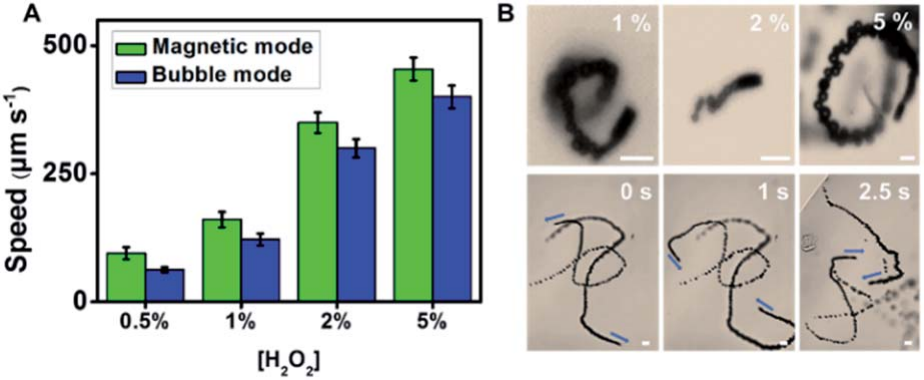
Tailored propulsion of WS_2_/Ni/Pt micromotors. (A) Speed dependence on peroxide concentration both in the absence (“*bubble-mode*”) and presence (“*magnetic mode*”) of a magnetic field. (B) Time-lapse images (taken from Video S1†) of micromotor propulsion at different peroxide concentrations in “*bubble-mode*” (absence of a magnetic field, top part) and “*magnetic mode*” (bottom part). Scale bars, 10 μm. Note the different lengths of the scale bar in the “*bubble-mode*” and “*magnetic mode*” images, which shows the longer trajectories for the latter case.

Interestingly, the observed granular structure of the catalytic layer of the as-synthesized micromotors in Fig. 1E (associated with the roughness of the WS_2_ layer) has a strong influence on the propulsion performance and catalytic activity of the inner layer (see Fig. 2). The inner catalytic Pt layer triggers the decomposition of the peroxide fuel, releasing oxygen bubbles from one side of the micromotors, which reached average speeds of 94 μm s^–1^ (0.5% H_2_O_2_) and 179 μm s^–1^ (1% H_2_O_2_). This speed is higher than that observed for WS_2_NPs–PANI/Pt and PANI/Pt micromotors, which propel at speeds of 71 and 39 μm s^–1^ (1% H_2_O_2_).^33^ Higher speeds were obtained for MoS_2_/Pt micromotors (370 μm s^–1^, 1% H_2_O_2_).^34^ However, it is worth noting here that the inclusion of Ni magnetic layers results in the decrease in the speed compared to the case where a Pt layer is included. To check this assumption, we prepared MoS_2_ micromotors with a Ni layer, and the speed decreased to 80 μm s^–1^ (1% H_2_O_2_). Compared with magnetic carbon-based nanomaterials containing a similar Ni layer, the speed of the WS_2_ micromotors was similar to that observed for micromotors containing carbon black (90 μm s^–1^) as the outer layer but slightly lower than that of multiwalled carbon nanotube based micromotors (200 μm s^–1^) propelling in 1% peroxide solution.^42^ As the micromotor contains an inner Ni layer, propulsion can be improved by the application of external magnetic fields. Interestingly, as displayed in Fig. 2A, at low peroxide levels (0.5 to 1%), the speed increases up to 60% after external control of the micromotor with a magnetic field. The corresponding time-lapse images are depicted in Fig. 2B. We attribute such a phenomenon to better fuel accessibility towards the catalytic layer, due to the specific orientation induced with the magnet, resulting in a larger exposed area for enhanced fuel decomposition. To check that such different speeds attributed to the motion in the linear and circular ways is not caused by different drag forces applied on the different part of the tubes, we studied a total of 50 micromotors in both modes and found a similar trend with no statistical differences. Numerical simulations (not shown) illustrate how the application of magnetic fields results in an increase of the overall speed, mainly due to a change in the flow regime (from turbulent to laminar) and better fuel accessibility. This increases the residence time and improves the contact of the fuel with the catalyst, resulting in a higher oxygen generation rate and an increase in the capillary force exerted by the generated bubbles. The effect is more pronounced as the level of fuel decreases. Yet, under unguided conditions, the fuel–inner wall contact is hindered, resulting in lower speeds. A similar behaviour was observed for MoS_2_/Ni/Pt and PEDOT/Ni/Pt micromotors. Thus, the speed of MoS_2_ and PEDOT based micromotors increased from 210 and 82 μm s^–1^ in non-magnetic mode to 265 and 140 μm s^–1^ in magnetic mode (from 1.3 to 1.6 times), similar to that observed for the as-synthesized micromotors. The numerical simulations describing such phenomena have been very recently described by our research group.^43^ From the above mentioned, it can be concluded that WS_2_ does not influence the peroxide decomposition activity. Yet, it can indirectly affect it by imparting an increased surface roughness to the inner Ni and Pt layers, as reflected by the increased speeds. This finding holds considerable promise for “*on-demand*” regulation of the micromotor movement. The efficiency of the micromotors under each condition (guided and unguided) was calculated (at 1% peroxide levels) following the model described by the Mallouk group.^44^ The efficiency of WS_2_ micromotors was found to be 2.40 × 10^–7^ and 2.60 × 10^–7^ in bubble and catalytically induced magnetic mode, respectively (see Table S1†). Such an efficiency is much higher than that previously reported for Pt-based tubular rolled-up micromotors (2.40 × 10^–10^, 3% H_2_O_2_)^45^ or even higher than that of C_60_ fullerene/Pt (1.44 × 10^–7^, 1% H_2_O_2_) and carbon-black–Pt (1.02 × 10^–7^, 1% H_2_O_2_) micromotors with a similar surface roughness and slightly lower than that of carbon nanotubes and graphene/Pt-based micromotors (from 3.28 to 4.92 × 10^–7^).^46^

**Fig. 3:**
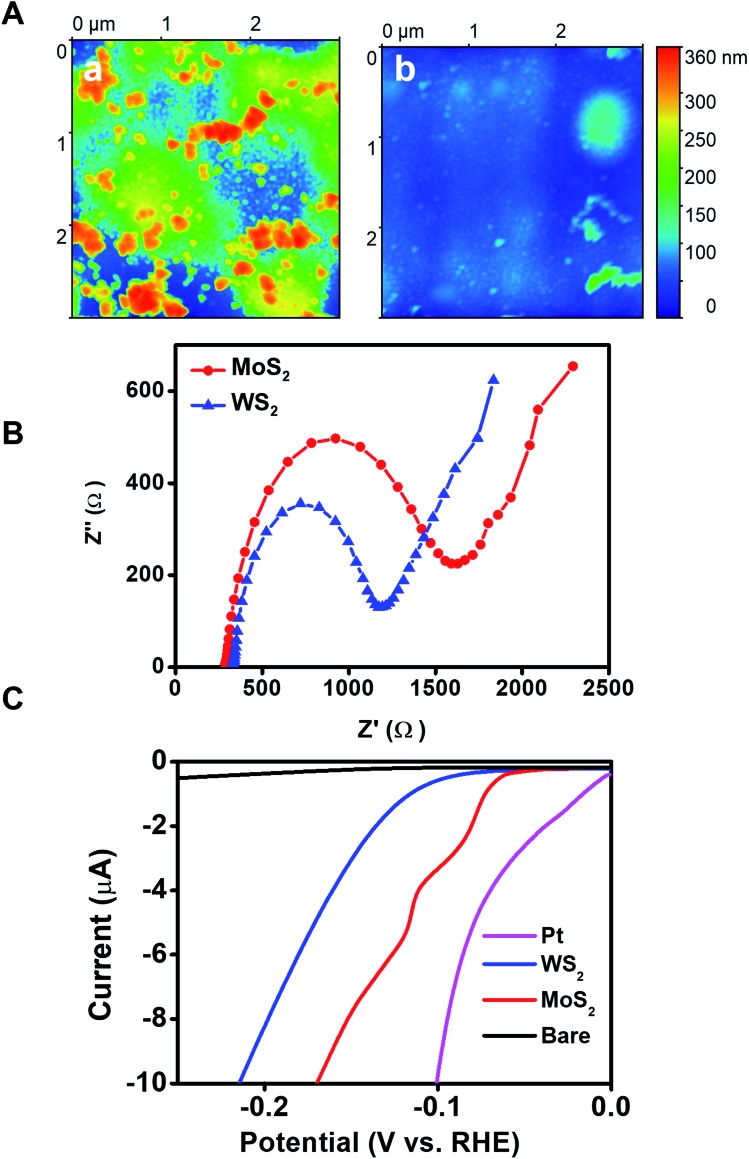
Surface morphology and catalytic activity of WS_2_/Ni/Pt micromotors. (A) AFM topographical images showing the surface morphology of (a) WS_2_/Ni/Pt and (b) MoS_2_/Ni/Pt control micromotors. Images were processed with Gwyddion software to subtract the micromotor curvature to better display the morphology at the nanometer level. (B) Nyquist plots and (C) linear sweep voltammograms of WS_2_/Ni/Pt and MoS_2_/Ni/Pt microtube-modified electrodes. For the Nyquist plots, Fe (CN)_6_^4–/3–^ is used as a redox probe.

The outer morphology and surface roughness of the WS_2_ micromotors were then characterized by atomic force microscopy (AFM). The topographical AFM images in Fig. 3A illustrate the rough and jagged outer morphology of WS_2_ based micromotors (mean roughness value = 28 nm), with a granular peak-and-valley type structure, with valleys indicated in blue-green color and protrusions in orange color. For comparison, we include similar TMD based control MoS_2_ (mean roughness value = 4.2 nm) micromotors, which interestingly are relatively smooth in comparison with WS_2_ ones (note the different vertical scales). Thus, the resulting outer exposed surface area possesses a high density of edges that are potential active sites, which can be very relevant for achieving enhanced electrochemical performance and sensing. Accordingly, in order to obtain further insights into the catalytic properties of the outer WS_2_ layer of the as-synthesized micromotors, we explore its potential for the hydrogen evolution reaction (HER). To this end, we modified different electrodes with WS_2_/Pt and MoS_2_/Pt control microtubes (indeed, since they are a static control, here they act as microtubes only). First, Nyquist plots in Fig. 3B illustrate that a lower charge transfer resistance was obtained for WS_2_ microtube electrodes (*R*_s_ = 302 Ω) as compared with MoS_2_ microtube electrodes (*R*_s_ = 350 Ω), indicating superior conductivity. Then we carried out HER measurements in 0.5 M H_2_SO_4_ using a standard three electrode configuration (for more details, see the Experimental section in the ESI†). For comparison, polarization curves of Pt and bare electrodes are also included, yielding similar values to those reported in the literature.^47^ The linear sweep voltammograms (LSVs) in Fig. 3C illustrate that the current response of WS_2_/Pt and MoS_2_/Pt at similar onset potentials toward hydrogen evolution (2H^+^ + 2e^–^ → H_2_) was greater than that of bare surfaces, although so far from that of Pt, as expected. Interestingly, such a result indicates that the inner catalytic layer in the WS_2_ microtubes still retains its catalytic properties for HER-based applications, holding considerable promise to reduce the high cost of such catalysts in these applications. Indeed, the high catalytic activity of WS_2_ microtube-supported platinum reflects the synergetic effect of the hybrid nanostructure, in which Pt provides highly active hydrogen adsorption sites and facilitates the charge transfer process for H^+^ reduction.^48^,^49^

**Fig. 4:**
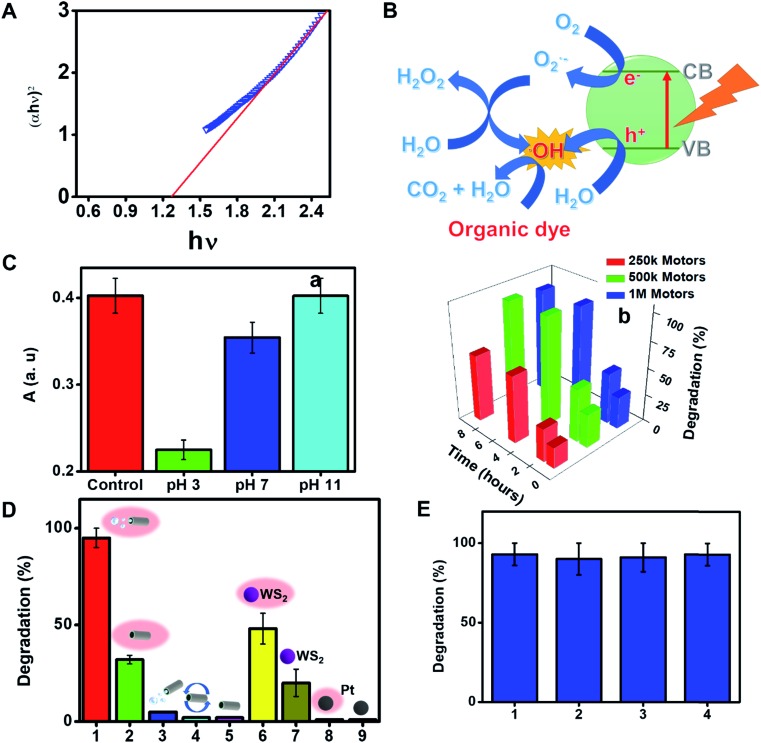
NIR responsive properties of WS_2_/Ni/Pt micromotors. (A) TAUC plot showing the bandgap of WS_2_/Ni/Pt micromotors. (B) Schematic of the removal of the model dye Remazol Brilliant Blue R (RBB) with the micromotors upon NIR light (800 nm) irradiation. (C) Effect of pH on RBB absorbance after photocatalytic treatment (a) and that of time and number of motors (b) on RBB removal efficiency. (D) RBB removal with WS_2_/Ni/Pt micromotors under different conditions: (1) moving under NIR light irradiation; (2) static under NIR light irradiation; (3) moving without NIR light irradiation; (4) under magnetic stirring; (5) static; (6) WS_2_ nanopowder under NIR light irradiation; (7) WS_2_ nanopowder without NIR light irradiation; (8) Pt nanoparticles under NIR light irradiation; (9) Pt nanoparticles without NIR light irradiation. (E) Reusability of WS_2_/Ni/Pt micromotors over 4 consecutive cycles.

The rich outer surface chemistry and the inherent photocatalytic properties of WS_2_ based micromotors were also exploited for “*on the fly*” NIR degradation of pollutants. First, the bandgap energy of the as-synthesized micromotors was investigated from the plot of (*αhν*)^2^*vs. hν* by extrapolating the linear portion of the plot to the *X* axis (see Fig. 4A) and was found to be ∼1.2 eV. Previous studies have reported that layered WS_2_ structures display NIR activity associated with the indirect bandgap (1.20–1.39 eV) coupled with its high valence band (1.17 eV *vs.* NHE).^7^,^50^ Thus, the micromotors possess a relatively high NIR photocatalytic activity which can be activated upon irradiation with wavelengths from 700 to 1000 nm (which correspond to photon energies from 1.77 to 1.24 eV). Upon interaction with NIR light, an electron (e^–^) will be promoted from the valence band to the conduction band in the WS_2_ outer micromotor layer; creating a hole (h^+^) in the valence band. The WS_2_ film promotes the rapid transfer of such electrons, inhibiting the recombination of photogenerated electron–hole pairs. The photogenerated electrons (e^–^) reduce adsorbed O_2_ molecules to generate O_2_^–^ radicals, whereas the holes (h^+^) in the valence band oxidize surface-absorbed water to produce OH^–^ radicals.^51^ Such photogenerated radicals along with the holes (h^+^) contribute to the photocatalytic degradation of dye pollutants and other organic compounds, as described in Fig. 4B. Such a fact holds considerable promise for novel environmental degradation mechanisms and for “*on-the-move*” water splitting applications.^52^

To further prove the concept and the NIR light photocatalytic activities of the as-synthesized WS_2_ micromotors, we examined their ability for the degradation of the model pollutant Remazol Brilliant Blue R (RBB). To this end, we designed a photocatalytic chamber (see the Experimental section for more details and a picture) integrating a 50 W NIR lamp with maximum emission at 800 nm. We first studied the effect of pH on the rate of photocatalytic degradation. Thus, 1 mL of water samples at different pH values contaminated with RBB (*C*_o_ = 35 mg L^–1^) was adjusted to the desired pH and added to a microcentrifuge tube containing the micromotors (2.5 × 10^5^ micromotors per mL), 1% SDS and 1% H_2_O_2_. Next, the tube was placed in the photocatalytic chamber for 120 min. The extent of RBB degradation was monitored by the decrease in its typical absorbance peak at 590 nm. As shown in Fig. 4C, pH 3 was optimal for degradation, with the highest degradation rates. At acidic pH, RBB is present in the anionic form, whereas the excess of H^+^ ions in solution induces a positive charge on the catalyst/micromotor surface. Thus, RBB adsorbs on its surface, where the positive holes (h^+^) can directly oxidize it. Furthermore, e^–^ can oxidize the oxygen adsorbed on the surface of the micromotors and promote the formation of radicals, resulting in additional RBB degradation.^53^ We next evaluated the effect of time and the number of micromotors upon degradation. Degradation rates increase as the number of motors and time increase up to 5 × 10^5^ micromotors per mL (which corresponds to 288 μg of micromotors) and 120 min, respectively, then reaching a plateau. Under the optimized conditions, several control experiments were performed. Remarkably low (30%) or negligible degradation rates were obtained using either static micromotors upon NIR light irradiation (in the absence of peroxide fuel) or magnetically stirred micromotors. We also evaluated the effect of pristine WS_2_ on micromotor degradation under the same conditions evaluated for the moving micromotors. The bandgap of such a material ranges from 1.4 to 2.0 eV. Commercial nanoparticles have a diameter of 90 nm (for additional SEM morphology, see Fig. S4†). As can be seen in Fig. 4D, degradation rates of 48 and 20% were obtained with and without NIR light irradiation, respectively. Such percentages are much lower than those obtained for moving micromotors but slightly higher than those observed for static micromotors. Such differences may arise from the difficulties in using the same amount of micromotors/particles. Yet, overall, such results reveal the inherent photocatalytic properties of the as-synthesized micromotors under NIR light irradiation and the crucial role of micromotor movement upon enhanced fluid mixing in improved degradation efficiencies. Additional control experiments were performed to address the role of peroxide and potential induced thermal effects in RBB degradation. To check the influence of H_2_O_2_ on RBB degradation, we performed control experiments in solutions containing the dye, 5% SDS, 1% H_2_O_2_ and Pt nanoparticles in the absence and presence of light. Negligible degradation was noted in all cases, revealing the negligible effect of peroxide on RBB degradation. Additionally, experiments were also conducted in the absence of Pt nanoparticles, with negligible degradation rates (not shown). In the case of Pt nanoparticles (which do not contain a bandgap for subsequent radical generation) negligible degradation was noted both in the presence and absence of light, indicating also the negligible effect of the local temperature on the degradation efficiency. UV-vis spectra in Fig. S3† clearly show the decrease of the 590 nm peak intensity with time. In the by-product spectrum, the 630 nm peak disappears, which, along with the red-shift and band broadening, indicates the breakup of the highly conjugated RBB system. Data were further processed under pseudo-first-order and pseudo-second-order degradation kinetic models (see the Experimental section for more details). As can be seen in Fig. S5,† RBB photocatalytic degradation obeys first-order kinetics, characterized by the Langmuir–Hinshelwood model (saturation kinetic mechanism). The slope of the plot of ln(*C*_0_/*C*) *vs.* time is 0.020 min^–1^, which is 2-fold higher than that reported for pristine WS_2_ (0.010 min^–1^) and 6-fold higher than that of WS_2_@MoS_2_ (0.0030 min^–1^), indicating the superior photocatalytic performance of the as-synthesized micromotors.^54^ Finally, the magnetic properties of the as-synthesized micromotors allow for complete recovery after solution treatment for subsequent reusability. Excellent performance is observed in the reusability test during four consecutive cycles, with almost 100% degradation rates in all cases.

## Experimental

### Equipment and reagents

Tungstic acid (Cat. no. 223328), sodium tungstate dihydrate (Cat. no. 223336), sodium sulfate (Cat. no. 238597), nickel(ii) sulfamate (Cat. no. 262277), nickel(ii) chloride (Cat. no. N6136), chloroplatinic acid hydrate (Cat. no. 254029), ammonium molybdate tetrahydrate (Cat. no. 431346), tungsten disulfide nanopowder (Cat. no. 790583) and Remazol Brilliant Blue R (Cat. no. R8001) were purchased from Merck (Madrid, Spain). Boric acid (Cat. no. 15665) and sodium dodecyl sulfate (Cat. no. 71727) were supplied by Fluka (Bucharest, Romania). All reagents were used without further purification. Template-assisted electrochemical deposition and electrochemical measurements were carried out using an Autolab PGSTAT 12 (Eco Chemie, Utrecht, The Netherlands). Scanning electron microscopy (SEM) and energy-dispersive X-ray mapping (EDX) images were obtained with a JEOL 6335 F microscope (JEOL USA, Massachusetts, United States) coupled with an Xflash detector 4010 (Bruker, Massachusetts, United States). The acceleration voltage was set at 20 kV for imaging and at 22 kV for EDX mapping. The AFM measurements were performed with a Nanoscope IIIa (Veeco) system operating in dynamic mode. Silicon cantilevers with a nominal radius of curvature of 8 nm and a spring constant of 40 N m^–1^ were employed. The samples were prepared by depositing a water drop containing the micromotors on a freshly cleaned mica surface. After drying, the sample was mounted on the AFM sample holder and the tip was addressed, thanks to the attached optical microscope, to a given micromotor. Once the tip reached the surface of the micromotor, it scanned the surface with the *x*-axis parallel to the longest axis of the micromotor. Otherwise, the tip, albeit operating in dynamic mode, was able to displace it. At least six micromotors were measured for each preparation condition. Raman spectra were recorded using an Alpha 300 AR (WITec, Ulm, Germany) confocal Raman microscope. The laser wavelength was 532 nm. All movies were recorded with a Zyla cMOS camera attached to an Eclipse Ti-S inverted microscope (Nikon, Tokyo, Japan).

### Micromotor synthesis and characterization

The electrochemical deposition takes place into 5 μm diameter conical pores of a polycarbonate membrane (Catalog no. 7060-2513; Whatman, New Jersey, USA). First, a thin gold film is sputtered on the branched side of the membrane to serve as a working electrode (a Pt wire is used as the counter electrode). To prevent gold from reaching the pores of the membrane, this was placed on a glass slide with the pore side facing the glass slide. Then, the membrane is assembled in a Teflon plating cell with aluminum foil serving as an electrical contact for the subsequent deposition steps. In this case, the exposed pores in the membrane were facing the plating solution. The first WS_2_ layer was electrodeposited from a solution containing H_2_WO_4_ (0.135 M), Na_2_WO_4_ (0.135 M) and Na_2_SO_3_ (0.18 M, S^2–^ precursor) at pH 9.2 by cyclic voltammetry (0.3 V to –1.2 V *vs.* Ag/AgCl (3 M), at 50 mV s^–1^ for ten cycles) (step a in Fig. S1†). The nickel-plating solution contains nickel sulfamate (1.2 M), nickel chloride (82 mM) and boric acid (464 mM) and is set to pH 4. The deposition of the nickel layer is performed by galvanostatic voltammetry in two steps: ten 100 ms pulsated nucleation scans (–20 mA) and a 300 s deposition scan (–6 mA). Finally, the platinum plating solution contains 4 mM H_2_PtCl_6_ in 0.5 M boric acid. Platinum is amperometrically deposited at –0.4 V for 750 s (step b and c in Fig. S1†). Once the deposition is finished, the gold layer is gently removed by hand polishing with 0.05 μm alumina slurry and the membrane was then dissolved in methylene chloride to completely release the microtubes. The micromotors were collected by centrifugation at 7000 rpm for 3 min and washed with isopropanol, ethanol and three times with ultrapure water (18.2 Ω cm), with a 3 min centrifugation step following each wash (step d in Fig. S1†).

### Micromotor counting

To estimate the number of micromotors in a batch, we first measured the area of a 1 μL drop (*n* = 10) as *A* = *πr*^2^. Then, we used a higher magnification objective and counted the motors in a predefined area. The amount of motors in this area was then extrapolated to the whole drop. The results were expressed as motors mL^–1^. The volume of micromotor solutions was adjusted (either by centrifugation or supernatant removal) to have a constant micromotor number.

### Electrochemical measurements

Linear sweep voltammograms were recorded on a carbon electrode modified with an identical mass of each micromotor or by depositing a Pt layer with a scan rate of 20 mV s^–1^ in 0.5 M N_2_-saturated H_2_SO_4_. The reference electrode was Ag/AgCl and all potentials were referred to the reversible hydrogen electrode. Current densities were calculated taking into consideration the geometrical area of the electrode. Electrochemical impedance spectroscopy (EIS) was carried out using K_3_[Fe(CN)_6_]/K_4_[Fe(CN)_6_] (1 : 1) (5 mM) in 0.1 M KCl or [Ru(NH_3_)]Cl_3_ and [Ru(NH_3_)]Cl_2_ (5 mM) in 0.1 M KCl solution and starting at open circuit potential using AC signals of amplitude 5 mV peak-to-peak in the frequency range of 100 000 to 0.01 Hz. The electrodes were modified by drop casting the micromotors.

### Micromotor propulsion experiments

Propulsion experiments were performed on glass slides over the microscope objectives. 1 μL of water-dispersed micromotors was mixed *in situ* with 1 μL 15% SDS and 1 μL of the different H_2_O_2_ solutions. Videos were recorded upon the addition of the fuel solution. The movie rate is set at 25 FPS and micromotor speed is tracked using NIS Elements AR 3.2 software (Nikon, Tokyo, Japan). Sodium dodecyl sulphate (SDS) is used as a surfactant in all propulsion experiments.

### Degradation experiments

Remazol Brilliant Blue (*C*_o_ = 35 mg L^–1^) samples were prepared in ultrapure water (18.2 Ω cm). Furthermore, 5% SDS and 1% H_2_O_2_ were added to allow micromotor propulsion. All solutions were adjusted to the desired pH, and then, each solution is added to a microcentrifuge tube containing dried out micromotors (the number of micromotors depends on the experiment). Photodegradation experiments are carried out in a custom-made photocatalysis cell (see Fig. S2†). The degradation cell is composed of an aluminum foil box, a 50 W IR light bulb (ExoTerra, Montreal, Canada) and a sample holder placed at 10 cm from the light source. The samples were monitored by measuring the decrease of the absorbance band (*λ*_abs_ = 580 nm). For reutilization experiments, micromotors were pulled aside using a magnet and the supernatant is pipetted gently.

Pseudo-first-order kinetics were calculated according to the equation:
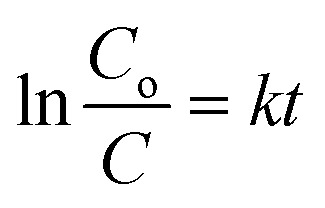
where *C*_o_ and *C* are the concentration of each pollutant in solution initially and at any time *t*, *k* is the reaction rate constant and *t* is the specific time. The model was evaluated by plotting ln(*C*_o_/*C*) *versus* time.

Pseudo-second-order kinetics were expressed as:
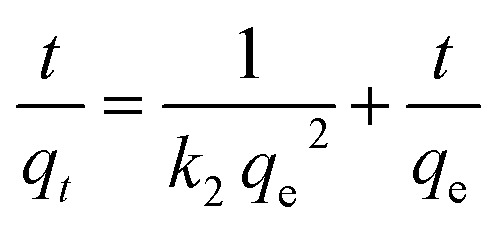
where *k*_2_ is the reaction rate constant and *q*_e_ and *q*_*t*_ refer to the amount of pollutant present at equilibrium and at time *t*, respectively. The amount of adsorption at time *t*, *q*_*t*_, was calculated using:
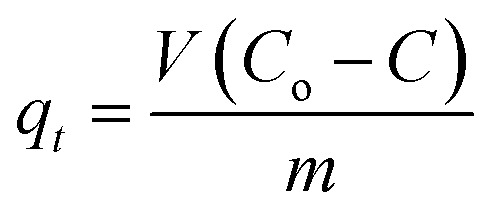


*C*_o_ and *C* are the concentration of each pollutant in solution initially and at any time *t*, respectively. *V* is the volume of the solution (1 mL) and *m* is the mass of micromotors used (280 μg). The model was evaluated by plotting *t*/*q*_2_*versus* time.

## Conclusions

We have illustrated the template electrosynthesis of micromotors composed exclusively of WS_2_ in the outer layer with highly reactive inner layers. Most importantly, the synthesis is performed at room temperature using tungstic acid and sulfate as cost-effective metal and sulfur source precursors. The resulting micromotors combine the excellent electronic properties of 2D chalcogenides (outer layer) with enhanced micromotor movement (inner layer). The combination of such dual capabilities in a single structure endows the micromotors with simultaneous “*inner and outer*” electrochemical and photo-enhanced electron transfer capabilities which facilitated unique applications of micromotors for hydrogen generation and environmental remediation schemes. The use of the inner Pt patch supported on WS_2_ still retains its catalytic properties for HER-based applications, holding considerable promise to reduce the high cost of such catalysts in these applications, where WS_2_ entirely constitutes the micromotor scaffold, compared with similarly prepared catalytic materials. These findings open new avenues for the use of micromotors as a moving catalyst in future “*on-the-move*” energy generation schemes. Yet, future efforts in this direction should be directed to increase such overall performance. The outer WS_2_ layer endows the micromotors with NIR responsive properties, which in connection with the autonomous movement associated with the inner layer results in the enhanced degradation of the model pollutant Remazol Brilliant Blue R. The ability to increase the micromotor speed by the application of a magnetic field (associated with a larger exposed catalytic surface area) can lead to better fuel decomposition, which can further enhance fluid mixing for improved electro or photocatalytic performance in further applications. We envision many more applications associated with the rich “*outer and inner*” chemistry of the as-synthesized micromotors, only limited by our imagination.

## Conflicts of interest

There are no conflicts to declare.

## Supplementary Material

Supplementary information

Supplementary movie
