# Supplementary material for: Near infrared-light responsive WS2 microengines with high-performance electro- and photo-catalytic activities
Source: Chem Sci. 2019 Oct 28;11(1):132–40. doi: 10.1039/c9sc03156a (PMC7012050; doi:10.1039/c9sc03156a)
Supplement: Supplementary file 1 [file SC-011-C9SC03156A-s001.pdf]

Electronic Supplementary Material (ESI) for Chemical Science.
This journal is © The Royal Society of Chemistry 2019

Electronic Supporting Information (ESI)

Near Infrared-light responsive WS_2 microengines with high performance electro and photo catalytic activities

Victor de la Asunción-Nadal,[a] B. Jurado-Sánchez,*[a, b] L. Vázquez[c] and A. Escarpa*[a, b]

[a]*Department of Analytical Chemistry, Physical Chemistry and Chemical Engineering, University of Alcala, Alcala de Henares E-28871, Madrid, Spain. E-mail: beatriz.jurado@uah.es; alberto.escarpa@uah.es (Tel.: +34 91 8854995).*

[b]*Chemical Research Institute "Andrés M. del Río", University of Alcala, Alcala de Henares E-28871, Madrid, Spain.*

[c]*Materials Science Factory. Institute of Materials Science of Madrid (ICMM-CSIC), Cantoblanco, E-28049 Madrid, Spain.*

Supporting videos

SI Video 1. Propulsion and magnetic guidance of WS_2/Ni/Pt micromotors in 1 % H_2O_2 solutions.

Supporting figures

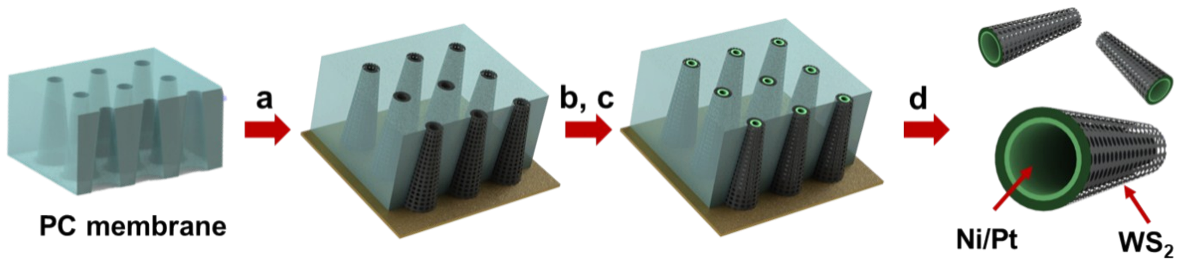

Fig S1. Schematic of the template electrodeposition of WS_2/Ni/Pt micromotors. a) WS_2 layer deposition by cyclic voltammetry; b, c) Electrodeposition of the inner magnetic Ni and catalytic PtNPs layer; d) Release of the microtubes from the membrane template.

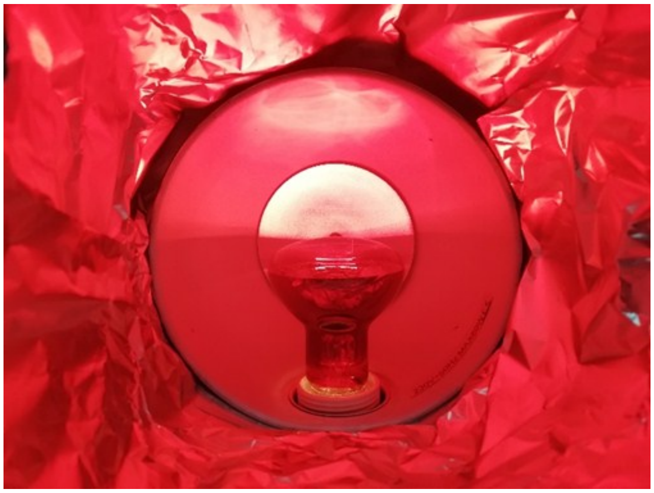

Fig S2. Inner view of the photodegradation chamber.

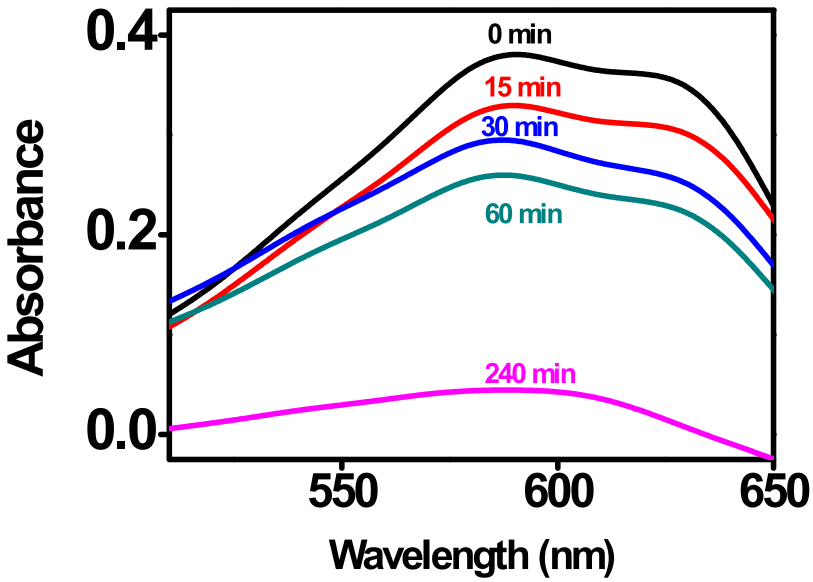

Fig S3. UV-VIS spectra of the degradation of Remazol Brilliant Blue R over time under the optimized conditions: 2.5 x 10^5 micromotors mL^{-1}), 5% SDS and 1% H_2O_2.

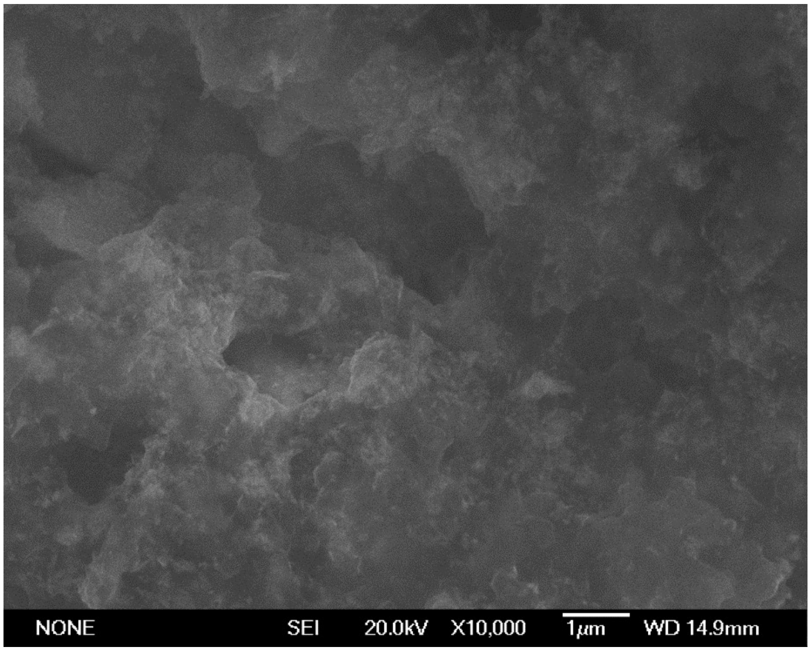

Fig S4. SEM characterization of the commercial WS_2 nanoparticles used as control to check RBB degradation.

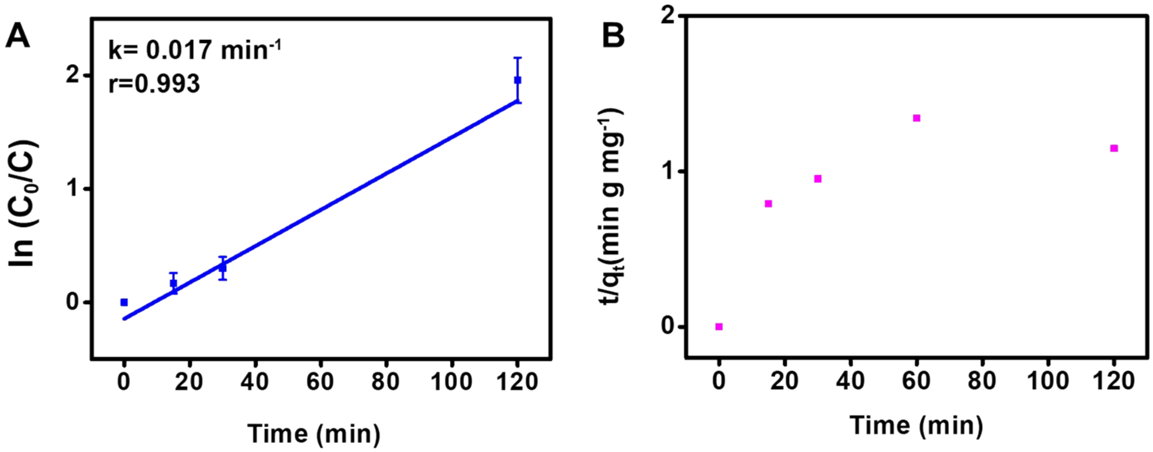

Fig S5. Pseudo-first order (A) and pseudo-second order kinetics (B) models for Remazol Brilliant Blue R degradation. Conditions: 2.5 x 10^5 micromotors mL^{-1}), 5% SDS and 1% H_2O_2.

Table S1. Calculation of efficiency of micromotors

	WS_2 bubble mode	WS_2 Magnetic mode
Bubble diameter, µm	7.26	8.22
Bubble rate, B_r (bubbles per second)	40	41
Volume of oxygen generated per second, m^3,$V=B_r\times4\pi r^3/3$	8.0×10^{-15}	1.2×10^{-14}
Oxygen evolution rate, mol/(motor·s), n=PV/RT	3.3×10^{-13}	4.9×10^{-13}
Chemical input power, W/motor, $P_{chem}= n\Delta_r{}^{\theta}G$	6.8×10^{-8}	1.0×10^{-7}
Average micromotor diameter, µm	5	5
Average micromotor length, µm	11	11
Average Micromotor speed, µm/s (1 % H_2O_2)	120	150
Mechanical power output, $P_{mecha}= fv^2=6\pi\mu rv^2$	1.6×10^{-14}	2.6×10^{-14}
**EFFICIENCY, P_{mecha}/ $P_{chem,}$ 10^{-7}**	**2.4**	**2.6**
